# Vascularisation of pluripotent stem cell–derived myocardium: biomechanical insights for physiological relevance in cardiac tissue engineering

**DOI:** 10.1007/s00424-021-02557-8

**Published:** 2021-04-14

**Authors:** Oisín King, Ilona Sunyovszki, Cesare M. Terracciano

**Affiliations:** National Heart & Lung Institute, Imperial College London, Hammersmith Campus, ICTEM 4th floor, Du Cane Road, London, W12 0NN UK

**Keywords:** IPSC, Pluripotent stem cells, Endothelial cells, Cardiomyocytes, Biomechanics, Mechanosensation, Models of disease, Organ-on-chip, Bioprinting

## Abstract

The myocardium is a diverse environment, requiring coordination between a variety of specialised cell types. Biochemical crosstalk between cardiomyocytes (CM) and microvascular endothelial cells (MVEC) is essential to maintain contractility and healthy tissue homeostasis. Yet, as myocytes beat, heterocellular communication occurs also through constantly fluctuating biomechanical stimuli, namely (1) compressive and tensile forces generated directly by the beating myocardium, and (2) pulsatile shear stress caused by intra-microvascular flow. Despite endothelial cells (EC) being highly mechanosensitive, the role of biomechanical stimuli from beating CM as a regulatory mode of myocardial-microvascular crosstalk is relatively unexplored. Given that cardiac biomechanics are dramatically altered during disease, and disruption of myocardial-microvascular communication is a known driver of pathological remodelling, understanding the biomechanical context necessary for healthy myocardial-microvascular interaction is of high importance. The current gap in understanding can largely be attributed to technical limitations associated with reproducing dynamic physiological biomechanics in multicellular in vitro platforms, coupled with limited in vitro viability of primary cardiac tissue. However, differentiation of CM from human pluripotent stem cells (hPSC) has provided an unlimited source of human myocytes suitable for designing in vitro models. This technology is now converging with the diverse field of tissue engineering, which utilises in vitro techniques designed to enhance physiological relevance, such as biomimetic extracellular matrix (ECM) as 3D scaffolds, microfluidic perfusion of vascularised networks, and complex multicellular architectures generated via 3D bioprinting. These strategies are now allowing researchers to design in vitro platforms which emulate the cell composition, architectures, and biomechanics specific to the myocardial-microvascular microenvironment. Inclusion of physiological multicellularity and biomechanics may also induce a more mature phenotype in stem cell–derived CM, further enhancing their value. This review aims to highlight the importance of biomechanical stimuli as determinants of CM-EC crosstalk in cardiac health and disease, and to explore emerging tissue engineering and hPSC technologies which can recapitulate physiological dynamics to enhance the value of in vitro cardiac experimentation.

**Supplementary Information:**

The online version contains supplementary material available at 10.1007/s00424-021-02557-8.

## Introduction

### Myocardial mechanics and multicellularity

The heart is a highly dynamic organ, promptly adjusting cardiac output on an on-demand basis. Coordination of this process requires myocardial cells to be attuned to external biochemical and biophysical feedback, which the body uses to inform the heart of current demand. Biomechanical stimuli determined by tissue stiffness and circulatory system pressures are essential regulators of CM function [94]. This regulation occurs on both acute timescales, as evidenced by the Frank-Starling response, and on chronic timescales as seen during disease-associated myocyte remodelling such as hypertrophy. However, biomechanical conditions also inform the behaviour of cardiac non-myocytes. Cardiac fibroblasts (FB) are well-characterised mechanosensors, with non-physiological mechanical conditions being their cue for phenotypic transition to myofibroblast, and initiation of disease-associated fibrosis [155]. It has also been shown that FB activation is mechanically regulated in a number of modalities, with distinct responses induced via changes in substrate stiffness, altered mechanical stimulation from beating CM, and via paracrine signalling when CM experience non-physiological substrate stiffening [60]. CM-FB mechanical communication has therefore enjoyed much discussion in recent years due to important implications for disease pathogenesis [150, 160] and potential design of novel therapeutic strategies [44, 141]. However, much remains unknown about the influence of dynamic biomechanical stimulation in contractile myocardial tissue on the behaviour of the most abundant cell type in the heart, the microvascular endothelial cell (MVEC). As ECs are highly adept mechanosensors [37], and critical regulators of myocardial function [20], it is likely that fluctuating biomechanical stimuli actively regulate the phenotype of myocardial MVECs. The importance of understanding this dynamic is apparent when one considers the extent of deviation from healthy biomechanical conditions seen in various cardiac diseases [193], such as fibrotic remodelling after MI, increased LV pressure during hypertension, or LV stiffening during heart failure (HF). How these pathological states and their associated biomechanical alterations affect the ability of MVECs to perform their functions, including the regulation of myocardial contractility, is currently unknown. However, coronary microvascular dysfunction (CMD) (other pseudonyms being “cardiac syndrome X” and “microvascular angina”) is becoming increasingly recognised as a distinct and prevalent driver of cardiac pathology [16], one whose diagnosis is complicated and for which treatment options are limited [32]. As the coronary tree descends into the myocardium from artery (> 400 μm) to arteriole (400–10 μm) to capillary (< 10 μm), narrowing vessel diameter confers a transition from high capacitance to high resistance. As such, the minimal force required for myocardial perfusion is determined by microvascular tone, which itself is dynamically informed by the metabolic requirements of the myocardium, ensuring an appropriate balance between blood supply and demand. In CMD, structural remodelling including reduction in capillary diameter, fibrosis, and capillary rarefaction [123], alongside functional endothelial dysregulation such as impaired nitric oxide [13] endothelin-1 (ET-1) related vasodilatory capacity [117] elevates resistance to the point of inhibiting flow, causing ischemia. This condition is present in 50% of chronic heart and coronary syndromes, but often also occurs in the absence of other local causes of myocardial ischaemia such as diabetes [61, 134], hypertension [7, 50, 169, 189], and dyslipidemia [57, 152, 208]. This ubiquitous nature of CMD has made the biomechanical and/or biochemical drivers of its aetiology difficult to tease out thus far.

### Obstacles in studying myocardial microvasculature

It is no coincidence that there exists a lack of understanding of myocardial MVEC biology, and limited diagnostic options for assessing the myocardial microvasculature in the clinic. Both stem from the technical difficulty associated with observing micro-physiological structures embedded in the densely crowded and mechanically dynamic beating myocardium. With a spatial resolution of 500 µm, it is not possible to visualise the cardiac microvasculature via angiography. Consequently, the diagnosis of CMD is typically made indirectly via process of elimination, i.e. abnormal coronary flow reserve and/or microvascular resistance despite angiographically normal arteries [140]. Additionally, the protocol required to make such a diagnosis is non-trivial, requiring invasive angiography and intracoronary perfusion of acetylcholine and nitrates to determine vasoreactivity and vasospasm [40, 41, 139]. Ongoing development of more specialised imaging techniques, such as cardiac magnetic resonance [106] and contrast echocardiography [82], can now directly determine whether microvasculature is perfused or not, and therefore represent significant diagnostic promise. However, these methods are insufficient to observe dynamic events at the cellular and molecular level, and are therefore unlikely to further our understanding of cardiac microvascular function at the level of basic biology.

A key question that therefore needs to be addressed is which models are best to investigate the cardiac microvasculature in a physiologically relevant way, without sacrificing experimental capability? Animal models have enabled considerable investigation of the biomolecular context surrounding CMD, as recently reviewed in depth by Sorop et al. [177]. CMD caused by chemical- or dietary -induced metabolic disorders in dogs, pigs, and rodents, have helped to understand some core mechanisms associated with dysfunctional myocardial perfusion, such as the disruptive roles of reactive oxygen species (ROS) [81], and renin-angiotensin activation [213] in endothelial vasoregulation, and sensitisation of coronary alpha-adrenoreceptors during sympathetic nervous activation [33]. However, due to anatomical, metabolic, and genetic inconsistencies between animals and humans, choosing an appropriate animal model for a given experimental question is not always possible. In vivo models also present the same observational difficulties as the clinic. Currently, the best described method for direct visualisation of biomechanics within the living cardiac microvasculature is transillumination of a stabilised portion of exposed myocardium, first described by Tillich et al. in 1971 as a means to quantify intra-microvascular red blood cell velocity [183], and later adapted by Nellis et al. in 1981 to assess vessel pressure and diameter [130]. While providing valuable early insight into the nature of cardiac microvascular biomechanics, this technique was not widely adopted, likely due to technical complexity, restriction to animal epicardium, and limited potential overlap with other quantitative experimental methods. As a result, development of in vitro model of vascularised myocardium with cellular resolution, physiological biomechanics, and human biology remains an ongoing challenge for cardiac experimental research. Yet, such a model is required to reveal fundamental mechanisms which regulate myocardial microvasculature biology and pathophysiology.

### Convergence of tissue engineering and stem cell biology to model myocardial microvasculature

A new strategy to tackle this challenge is now emerging, as recent developments in stem cell biology [3, 209] and tissue engineering [86, 109] have established the groundwork for production of human in vitro cardiac models with unprecedented biomechanical relevance. Differentiation of cardiomyocytes from human embryonic stem cells (ECS-CM) or induced pluripotent-derived stem cells (iPSC-CM) is a well-established method of modelling human cardiac biology in vitro. This technique allows researchers to generate limitless numbers of beating CM with human genotype. These cells can be patient-specific, disease-specific, and genetically modified without ethical complication, making them a uniquely powerful tool for cardiac experimentation. While cardiac myocytes obtained from differentiation of hPSC exhibit important functional differences to primary adult CM [77], their in vitro generation bestows superior viability in the dish when compared to the rapid de-differentiation of isolated primary CM [132]. This longevity makes hPSC-CM a versatile candidate for modelling cardiac phenomena which occur on timescales of more than a few hours or days. Their phenotype is considered “immature”, and more closely represents that of a neonatal CM, whose gradual development into a mature myocyte occurs after birth in the beating myocardium [180]. hPSC-derived cardiomyocytes (PSC-CM) also exhibit considerable plasticity, encouraging their use in in vitro platforms which require adaptation to a new environment, such as encapsulation in 3D biomaterials [217], combination with additional cell types [196], and exposure to biomechanical conditioning [164]. Such models drive the phenotype of PSC-CM towards that of a mature adult CM, illustrating the advantage of their plastic nature and the beneficial consequence of replicating in vivo microenvironmental conditions. Meanwhile, vascular tissue engineering models have also been advancing in biological relevance, with “organ-on-a-chip” and 3D bioprinting technologies giving rise to numerous effective protocols of creating perfusable macro- [89] and microvasculature [128] in vitro [120]. By combining functionally perfusable vessels with tissue- or disease-specific cell types, many biological microenvironments (including cardiac) have been recreated in vitro for basic biology, disease modelling, and drug screening applications [142]. Additionally, strategies which recapitulate physiologically relevant biomechanical stimulation, such as rhythmic mechanical actuation of cell culture chambers to mimic the cardiac cycle [115], are further enhancing the value of in vitro models [53].

This review therefore aims first to emphasise and explore the unique biomechanical conditions in which healthy and diseased cardiac microvasculature operates, such that novel in vitro platforms can prioritise physiological relevance. We then discuss emergent tissue engineering technologies which propose to unlock new insights into the fundamental nature of myocardial-microvascular communication.

## Biomechanical conditions in myocardial tissue

**Fig. 1:**
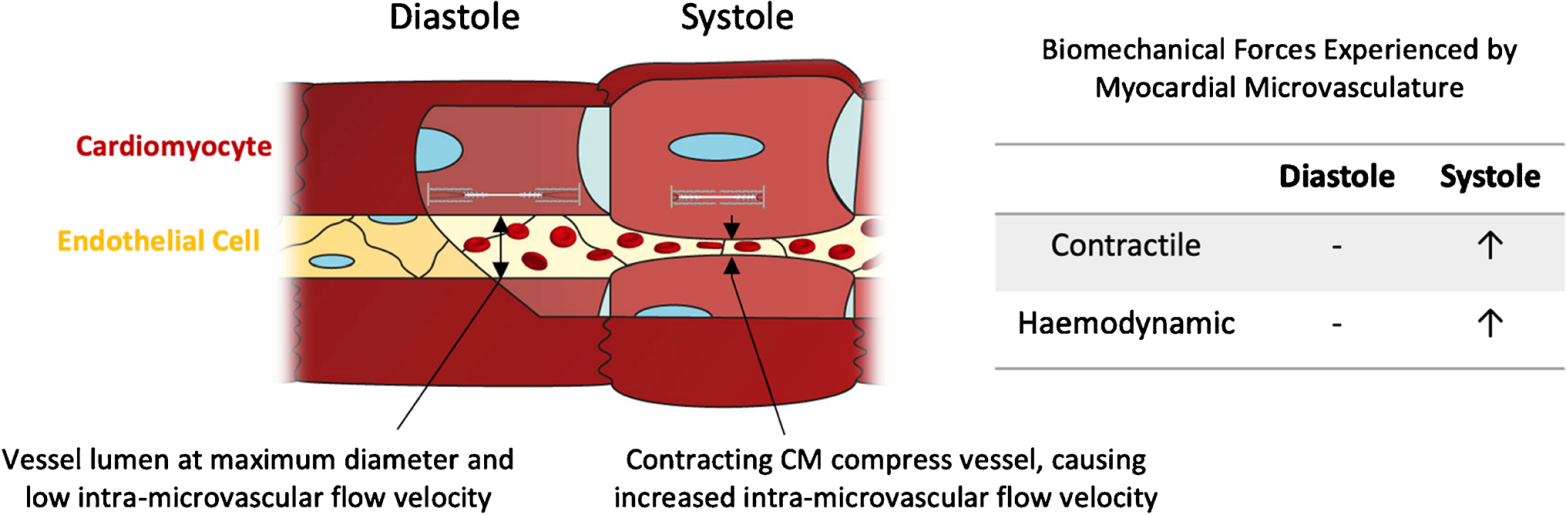
Biomechanical stimuli in myocardial-microvascular microenvironment during the cardiac cycle

In order to reproduce physiologically relevant biomechanical conditions in vitro, it is helpful to consider the organisation and orientation of different myocardial cellular structures. There are different types of myocardium within the human heart which are specialised to perform precise functions [79], but this review will focus on the compact myocardium, designed for force generation and found in abundance in the left ventricular free wall. Within the myocardium, distinct endothelial subtypes also exist. Myocardial EC subpopulations can be broadly divided into the endocardial endothelium (EE) which line the four chambers, and the microvascular endothelium (MVE), which comprises the capillaries permeating the myocardium [21] (Fig. 1). These subpopulations are distinctly cardiac as the ECs communicate directly with CMs, unlike the larger coronary vasculature in which ECs communicate with smooth muscle cells [20]. While EE cells also communicate directly with CM, the interaction between myocytes and MVECs represent the most widespread myocardial-endothelial interaction, and therefore would be a logical initial aim for in vitro modelling. In compact myocardium, CM fibres and capillaries are densely packed together in parallel, with all CM being in direct proximity to multiple vessels to enable bidirectional traffic of fluid, nutrients, and metabolites [21]. To sustain the metabolic activity required by the beating heart, the microvasculature permeates the myocardium at a density of 2000–3000 capillaries/mm, each with a mean diameter of 6.1 ± 1.2 μm [80]. The consequence of capillaries being only one cell thick, and lacking the smooth muscle and ECM barriers of their larger vessel counterparts, is that these ECs are mechanically coupled to neighbouring CMs. As such, the dynamic forces generated by beating myocytes over the cardiac cycle are felt by neighbouring ECs (Fig. 1).

### Extravascular biomechanics—contractility

During heartbeat, myocardial tissue deforms to expel blood from the ventricles, then relaxes to allow the chamber to refill. This deformation produces cyclical compressive and tensile forces, which are transmitted through the tissue via ECM proteins and cells. The importance of such intrinsic mechanical stimuli as regulators of heart function is an old concept in cardiology, one whose understanding extends back to the seventeenth century [94]. This idea, termed mechano-electric coupling, describes a relationship between myocardial biomechanics and contractility [87]. As CMs are the effectors of contractility, much work has been focussed on unravelling the molecular mechanisms through which they sense and respond to biomechanical stimuli [168]. Yet the same level of scrutiny has not been applied to ECs, meaning the consequence of contractile biomechanical stimuli on cardiac microvascular phenotype remains largely speculative [170].

During contraction, two models describe (in agreement) the mechanical deformation of the intramyocardial blood vessels: the time varying elastance model, and the muscle shortening and thickening model [198]. Via time varying elastance, left ventricular luminal pressure increases during systole, thereby causing an increase in intramyocardial pressure and stiffness, compressing the myocardial vessels and causing a decrease in vessel diameter [92, 93] (Supplementary Video 1). Similarly, via muscle shortening and thickening, as myocytes contract, they reduce their length (in parallel with capillaries) and increase their cross-sectional area (perpendicular to capillaries), also resulting in reduction in vessel diameter. If changes in coronary vascular volume are prevented, myocytes are unable to effectively shorten and contractility is impaired [201]. It should be noted that this relationship has been validated in myocardial arterioles and venules, but not capillaries due to technical limitations. However, Yada et al. reported almost identical diameter change profiles over the contractile cycle in both arterioles and venules of the porcine subendocardium, providing evidence that the relationship may be conserved in the intervening capillary network [203]. For both venules and arterioles, the reduction in diameter during systole varies throughout the myocardial wall, but is at a maximum of 30% reduction [198] (full obstruction does not occur).

How cyclic biomechanical stimulation specifically affects cardiac microvasculature is yet to be appreciated; however, a recent review by Fang et al. describes the in vitro and in vivo effect of cyclic biomechanical stimulation of ECs [37], with an aim of understanding how these stimuli might influence lung EC function (who experience biomechanics comparable to the heart as the lungs expand and contract during breathing). In brief, cyclic strain has been shown to regulate a variety of EC functions relevant to cardiac physiology and pathophysiology, including nitric oxide (NO) [179] and reactive oxygen species (ROS) [159] production, barrier permeability [17], inflammation [176], proliferation, and apoptosis [133]. Additionally, ECs use mechanical signals to regulate tissue morphogenesis [185]. Cyclic strain on EC regulates ECM remodelling [56], capillary orientation [182], and angiogenesis [127], meaning that co-culture with beating iPSC-CM may help induce a more biomimetic physical association in in vitro co-cultures. With this in mind, an ideal in vitro model would couple both iPSC-CM and EC in direct proximity (as they are in the human heart), such that mechanical forces can be transmitted bidirectionally between cells and globally through the tissue, allowing investigation of the (potentially significant) endothelial contribution to mechano-electric coupling. Models using biomimetic 3D substrates which can facilitate this dynamic will be discussed later in this review.

### Intravascular biomechanics—haemodynamics

In the wider context of mechanosensation, ECs are most commonly associated with shear stress caused by circulating blood flow [28]. However, shear stress within the microvasculature is often assumed to be negligible, due to high resistance and low flow rates [170]. While this may be true in most tissues, especially tissues distant from the heart, measurements made by transillumination of cat atrial muscle by Hellberg et al. in 1972 [58], and dog epimyocardium by Ashikawa et al. in 1986 [8] describe a pulsatile flow velocity profile within epimyocardial capillaries over the course of the contractile cycle. Ashikawa et al. reported a peak red blood cell velocity of 4 mm/s during systole and, to add a further layer of biomechanical complexity, retrograde flow of − 2 mm/s during diastole. The effect of flow direction reversal in ECs has been shown to influence many aspects of their behaviour [197], including production of cardioregulatory factors such inotropic agents (NO, ET-1) [137], growth factors (PDGF-A, FGF, TGF-a, TGF-b, VEGF, IGF-1, EGF) [147], barrier permeability [1], and inflammatory response [15]. Interesting differences in subepicardial and subendocardial arteriole flow profiles were documented by Toyota et al., who demonstrated dramatic retrograde systolic flow in subendocardial arterioles, contrasting with predominantly forward systolic flow in the epicardial vessels [188]. These differences are thought to contribute to higher susceptibility to ischaemia in the subendocardium [63]. This mismatch in flow velocity profiles is a phenomenon known as phase opposition of velocity waveforms, i.e. forward flow in the coronary arteries is predominately diastolic, whereas in coronary veins, it is predominately systolic. As a result, intramyocardial vessels act as an intermediary capacitance network, through which phase is swapped over the course of the contractile cycle, as reviewed in detail by Kajiya et al. in 2008 [75]. Kiyooka et al. observed this phenomenon at the level of the microvasculature by imaging coexistence of both diastolic and systolic flow-predominant epimyocardial capillaries [85]. Conserved pulsatile flow and wall shear stress down to the level of myocardial capillaries have also been predicted by mathematical modelling of the entire coronary arterial tree [69, 70]. Additionally, experimental evidence from Park et al. has shown that EC are sensitive to extremely low shear stress rates (10^−2^–10^−4^ dynes/cm^2^), testing values even lower than typically found in in vivo microcirculation (0.1 dynes/cm^2^) [146]. Under such low flow rates, EC display increased NO production and proliferation rates in the absence of cytoskeletal remodelling, when compared to static controls.

Shear stress of 15 dynes/cm^2^ caused by laminar flow was shown to increase and maintain EC expression of connexin-43 [31], the primary gap junctional protein that allows CM to propagate electrical current from cell to cell through the myocardium. The question of whether cardiac fibroblasts electrically couple to CM in the heart has been the subject of much debate in recent years [141]. However, even less is known about CM-EC electrotonic coupling. Given the abundance and widespread distribution of MVEC in myocardial tissue, existence of heterocellular electrotonic coupling has significant implications for cardiac physiology and pathophysiology. Connexin expression is known to be higher in EE cells (who experience high magnitudes of shear stress) compared to MVEC [20], which indicates subtype-specific phenotypes with regard to cell–cell communication. Taken together, these studies show that myocardial MVECs experience specific flow and shear stress conditions which are likely important in determining their behaviour, and therefore should not be overlooked in in vitro models which aim for physiological relevance.

### Polarisation and the importance of the lumen

For an EC to correctly perform its duties, it must be oriented appropriately, with a luminal surface exposed to blood flow and an abluminal surface in contact with extravascular cells and ECM (also known as apical/basolateral polarity). The luminal side experiences haemodynamic forces, i.e. shear stress from the blood flow, and interacts with erythrocytes, immune cells, neurohormonal factors, metabolites, and other circulating factors. Meanwhile, the abluminal side is tasked with communication with extravascular cells and interpretation of any intrinsic mechanical forces within tissue. These forces are transmitted through the ECM proteins and cells to which EC are anchored, which, in the case of the myocardial microvasculature, is predominantly a direct interaction with CM and collagen [43]. The differences in mechanical stimuli experienced on either side of the polarised EC are reflected by the mechanosensory apparatus localised on each side [37]. On their luminal surface, ECs express shear stress sensors such as mechanosensitive ion channels [46] and protruding cilia and glycocalyx, whose deflection allows the cell to respond to changes in blood flow [49]. On the abluminal side, expression of transmembrane integrins tether the EC cytoskeleton to the ECM via focal adhesion complexes [22], providing structural continuity through which external mechanical forces enter the cell [163]. Additionally, cell–cell junctional complexes which connect EC together, such as vascular endothelial specific cadherin (VE-cadherin) and platelet endothelial adhesion molecule (PECAM-1), act as both shear and compression/tensile sensors [35]. Mechanosensory proteins and their deliberate localisation are known to influence gene expression and molecular cascades in distinct and specific ways [37], thereby conferring microenvironment-specific properties to EC subpopulations. While much remains to be understood about myocardial MVEC mechanobiology, high-resistance arterioles and capillaries have long been known to be the point in the coronary tree where myocardial perfusion is regulated [143]. This essential process relies on EC being attuned to upstream biomechanics, meaning accurate modelling would require appropriate orientation.

Endothelial architecture is also important for CM function, as it forms a selectively permeable barrier which separates CMs from nutrients, biomolecules, cells, and other circulating factors [21]. In the heart, the EE displays deeper intracellular clefts (which permit molecular transport) and higher permeability when compared to the MVE, indicating more stringent regulation of transendothelial passage in the myocardial capillaries [6]. This physiological arrangement has also been proposed to dictate the modes of EC-mediated cardiac regulation via two processes: (1) stimulus-secretion coupling, where physical or circulating biochemical stimuli induce production and secretion of cardioregulatory factors in EC, and (2) the blood-heart barrier, in which the endothelium regulates the ionic homeostasis in the myocardial microenvironment via specific luminal-abluminal distribution of ion transporters [21]. While it is clear that this physical arrangement is necessary for normal function, these processes are poorly understood as they are difficult to interrogate in vivo; thus, establishment of an in vitro model of myocardial-microvascular interaction with a perfused, impermeable, and polarised lumen would allow valuable insights into EC cardio-regulation.

As apical/basolateral polarity and lumen formation are complex processes, they are often neglected in vitro for practicality. However, hemodynamic forces are important drivers of lumen formation [74], meaning endothelial polarity can be promoted in in vitro models via incorporation of physiologically relevant flow [84]. Tissue engineering techniques which recapitulate biomimetic haemodynamics and promote native vascular architectures will be discussed in the latter part of this review.

## Biomechanical regulation of CM-EC communication

Crosstalk between the microvasculature and myocardium regulates a diverse array of acute and chronic cardiac functions [20, 170], offering a promising target for therapeutic modulation of contractility or tissue remodelling [102]. Yet, while current knowledge of mechanisms underlying CM-MVEC communication is largely restricted to biochemical and paracrine signalling [184], maintenance of cardiac homeostasis also requires biomechanical feedback at the cellular and organ level [151]. Beating CM are therefore the source of mechanical stimuli which ultimately regulate themselves, both directly (they sense the changes in tensile and compressive forces in myocardial tissue during contraction) and indirectly via MVEC (who sense both contractile forces on their abluminal side and haemodynamic forces on their luminal side). This section aims to emphasise the current evidence for biomechanical stimuli as mediators of myocardial-microvascular crosstalk, which may inform in vitro model design.

### Biomechanical forces regulate CM-EC crosstalk in the developing heart

Healthy heart development requires a balance between biomechanical forces generated by myocyte contractility and blood flow [5, 30, 55, 105]. Auman et al. demonstrated that neonatal CMs transition from small, circular morphology, to the elongated rod-like morphology of the adult myocardium as a result of negative feedback from contractile forces, and positive feedback from haemodynamic forces [9]. While the shear stress within the endocardium was not specifically shown in this study to act via EC, or indeed in other studies which describe biomechanical regulation of myocyte hypertrophy [103, 205], it is highly likely, as EC-mediated shear stress is required for mature myocardial morphogenesis [34, 65, 192]. In the process of left ventricular trabeculation, CM contractility causes blood flow and activates shear sensitive cilia on the EE, which triggers a notch-neuregulin signalling cascade mediation of myocardial morphogenesis [167]. As such, forces generated by CMs instruct ECs to coordinate the growth of CMs. However, endocardial ECs also respond to increased tension in growing myocardium by proliferating, thereby ensuring parallel development of endocardium and myocardium [19]. Heterocellular and biomechanical feedback loops are therefore overlapping, making dissection of the relative contributions of each type of cell and/or biomechanical stimuli a challenging endeavour.

However, such feedback may also enhance the value of in vitro models. With respect to CM function, mechanical strain has recently been proposed as the coordinator of the embryonic heartbeat. In a model described by Chiou et al., coordination of the heartbeat in the soft microenvironment of the heart relies not on electrical communication from one CM to the next (as occurs in adult tissue), but in mechanical activation transmitted through the tissue [27]. This process is specific to the low stiffness of the embryonic heart. As the heart matures and stiffens over time, mechanical regulation gives way to the electric nature of adult tissue. Given the similarity between neonatal CM and iPSC-CM (particularly in relation to autonomous beating behaviour), this has significant implications for both maturation strategies of iPSC-CM and interpretation of iPSC-CM functional data in existing models. The mechanism which coordinates the stiffening of myocardial tissue in postnatal development is proposed to be a mechanical feedback loop between CM and FB [11, 110]. CM contractility sends mechanical signals to FBs, instructing them to produce stiffer ECM. As the tissue develops, heterocellular feedback ensures that it is continually of an optimal stiffness for myocyte contractility [111]. In this manner, force production increases, leading to enhanced blood flow and the previously discussed haemodynamic stimuli instructing EC to promote further myocardial growth. These mechano-cellular feedback loops illustrate a fine balance between biomechics and heterocellularity in cardiac development. By simultaneously increasing different types of biological complexity (heterocellularity and biomechanics) in vitro models, compounded benefit may be gained.

### Biomechanical forces regulate CM-EC crosstalk in cardiac homeostasis

CM-EC crosstalk is also necessary for regulation of homeostasis in the adult heart. Seminal work by Dirk Brutsaert and others identified paracrine communication from the endothelium to the myocardium as an acute modulator of contractility, chronotropy, inotropy, and lusitropy [20, 135, 170]. This homeostatic communication is also bi-directional, with CM secreting many vasoregulatory factors, including ET-1, NO, VEGF, and FGF2 [184].

Experimental investigation on the extent of biomechanical influence on CM-EC paracrine crosstalk is currently limited. However, a recent meta-analysis on expression of cardioregulatory protein expression in EC in hearts with disturbed loading conditions (acutely and chronically) reveals dramatic changes in a wide array of EC factors critical for maintenance of contractility and homeostasis, including NO, ET-1, neuregulin (NRG-1), prostacyclin, and angiopoietin 2 [170]. An ideal in vitro model would therefore recapitulate the physical coupling between CM and MVEC. In 2015, McCormick et al. demonstrated a relationship between CM contractility and EC mechanosensation via PECAM-1 (also known as CD31) [117]. PECAM^−/−^ mice displayed impaired diastolic and systolic function in vivo, without any observable differences in isolated CM in vitro. Compromised contractility was shown to be due to elevated endothelial NO/ROS and dysregulated NRG-1/erbb2 signalling. In these mice, EC have a reduced capacity to sense the mechanical stimuli of beating myocytes, which disrupts their normal paracrine signalling to myocytes and causes a loss of homeostasis, illustrating an overlap between physical and biochemical modes of crosstalk. However, the breadth of mechanosensitive [28, 37] and cardioregulatory [20, 170] signalling networks in EC alone indicates that we are only scratching the surface of this interaction. To decipher how hetero-cellular signalling pathways and molecular mechanisms overlap with physiological/biomechanical phenomena will likely require physiologically relevant in vitro models which combine functional and multi-omics analysis. Design of iPSC-based tissue engineering models which allow acquisition of such data would prove highly valuable.

However, not all interactions can be explained at the level gene expression. An interesting phenomenon, known as the Gregg effect, occurs when increases in microvascular volume enhances myocyte contractility and oxygen consumption [51]. The expansion of filling vessels deforms the membrane of neighbouring CMs, activating stretch-sensitive Ca^2+^ channels resulting in increased developed tension [95]. There also exists a relationship between coronary perfusion and diastolic force–length relationship, with increases in perfusion pressure causing increased diastolic stiffness [4]. While the Gregg effect is thought to be a largely mechanical process, as inhibition of NO [95] or the endothelial glycocalyx (a primary shear sensor) [165] does not reduce its effect, the mechanism regulating the relationship between diastolic pressure–volume and coronary vessel filling has been obscured by the technical difficulties associated with measuring coronary flow dynamics and/or shear stresses in vivo [198]. These phenomena illustrate the importance of accounting for physiology in in vitro studies.

### Biomechanical forces regulate CM-EC crosstalk in cardiac disease

A recent argument has been proposed for the reappraisal of ischaemic heart disease (IHD) terminology in order to reflect the growing clinical evidence that coronary microvascular dysfunction is a prevalent and distinct pathological state, often occurring in the absence of coronary arterial disease (CAD) [16]. A historical reliance on CAD as the driver of adverse outcomes has been called into question by the ISCHEMIA trial [114, 126], a study performed in stable IHD patients; this trial failed to show benefits of cardiac catheterisation and coronary revascularisation, as compared with non-invasive therapy. Consequentially, there is a growing focus on understanding microvascular phenotype in conditions such as ischaemia with no obstructive CAD [59] (INOCA) and myocardial infarction with no obstructive CAD [148] (MINOCA). A novel paradigm for heart failure with preserved ejection fraction (HFpEF) implicates coronary microvasculature dysfunction, induced by a systemic proinflammatory state, as the main driver of cardiomyocyte dysfunction [149]. This paradigm posits that systemic inflammation induces (1) coronary microvascular permeability (typically measured by increased expression of immune cell adhesion molecules) allowing transmigrating monocytes to initiate fibrosis, and (2) increased endothelial oxidative stress reducing NO bioavailability and cardiomyocyte cyclic guanosine monophosphate, ultimately leading to hypertrophy and myocardial stiffening [45, 123, 199]. As we have already considered, these are fundamental endothelial functions which are regulated by biomechanical stimuli [26]. The contribution of biomechanics to cardiac microvascular dysfunction and cardiopathogenesis remains an important open question, requiring dedicated investigation on relevant in vitro platforms.

Additionally, ECs play an active role in regulation of the myocardial biomechanical environment during response to disease. Recent single-cell sequencing of ECs in adult mouse hearts shows upregulation of genes related to cardiac remodelling and EC-ECM interaction in response to MI [101]. Endothelial-to-mesenchymal transition was also shown to contribute to pressure overload– [210] and MI- [187] associated fibrosis. Interestingly, in the reverse direction, mesenchymal-to-endothelial transition was observed to enhance neovascularisation after acute ischaemic injury [190].

The majority of cardiac disease is induced by environmental factors such as diet and smoking, and manifests over long time period. With an in vitro viability limited to a few days, healthy primary cardiac tissue is unsuitable for modelling chronic disease aetiology. However, with cardiac myocytes differentiated from human stem cells having an essentially unlimited in vitro lifespan, the door to clinically relevant timescales for experimental in vitro cardiac biology is now open. A perfusable model which recapitulates a polarised endothelium and underlying myocardium would be highly valuable in determining whether the microvasculature can be targeted for therapeutic modulation.

### Cardiac biomechanics to drive iPSC-CM maturity

We have examined the necessity of specific biomechanical stimuli to coordinate the cellular crosstalk through which the heart develops and functions. We have also touched on the immaturity of iPSC-CM, and the need to drive their phenotype towards that found in the healthy adult human heart for truly representative in vitro models. The obvious question then is whether incorporation of biomimetic mechanical stimuli in vitro can help promote the maturation that occurs in vivo? Based on current evidence, the answer would seem hopeful. A landmark proof-of-principle study by Cho et al. in 2017 showed that PSC-CM transplanted into the neonatal mouse heart matured and developed adult myocyte morphology, structure, and function after 1 month [29]. This work shows that given the ideal microenvironmental conditions, PSC-CM can become very similar to native CM, thereby establishing the goalposts and issuing a challenge to cardiac bioengineers the world over. Indeed, many studies before and since have shown that increasing the physiological relevance of either differentiation protocols or post-differentiation culture conditions can have a beneficial effect on iPSC-CM maturity [3]. Maturation can be assessed by a number of different (but ideally a combination of multiple) responses, such as ultrastructural remodelling (including sarcomeric apparatus organisation, sarcolemmal coupling to contractile machinery, and t-tubule generation), enhanced excitation–contraction coupling, metabolic shift from glycolysis to fatty acid oxidation, and transcriptomic/proteomic shift towards native adult CM profile [54]. Biomimetic maturation strategies investigated so far have included prolonging culture duration [76, 108], changing substrate stiffness [42] and orientation [158], 3D culture [99], metabolic conditioning [39, 64, 207], hormonal/biochemical conditioning [145, 206], incorporation of multicellularity [48, 78], and mechano-electric conditioning [161, 164]. While showing encouraging trends towards a more valuable human cell model, maturation is still in early phases and no standardised, widely accepted protocol has emerged. Additionally, all of the aforementioned methods have a positive influence on iPSC-CM maturity when applied individually, yet CM in vivo are subjected all of these conditions in combination. Maturation strategies and in vitro platforms which simultaneously account for the biomechanical and heterocellular dynamics required for cardiac development will likely further enhance maturity and are a logical next step for this field.

## In vitro models of myocardial-microvascular interaction to recapitulate physiological biomechanics

Having established the dependence of cardiac function on both myocardial-microvascular communication, and biomechanical stimuli, we can now turn our attention to in vitro strategies which may replicate these dynamics. It would be helpful at this point to establish some ideal characteristics for future models to promote more physiologically relevant cell behaviours:

1. **Multicellularity and cell–cell association**—CM and EC coupled in direct proximity (ideally in 3D) to enable biomimetic physical and paracrine modes of communication
2. **Mechanotransmission**—Substrate which facilitates cell deformation and propagation of mechanical stimuli, e.g. hydrogel/other non-rigid scaffold
3. **Haemodynamics**—Incorporation of controlled, biomimetic perfusion, exposing EC to flow
4. **Orientation/architecture**—Polarised EC with open lumen, continuous endothelium separating myocardium from nutrients/circulation/drug delivery, etc.

With this ideal “wish list” in mind, we can turn our attention to recent advances in tissue engineering which are enabling generation of such models.

### Static 3D co-culture

**Fig. 2:**
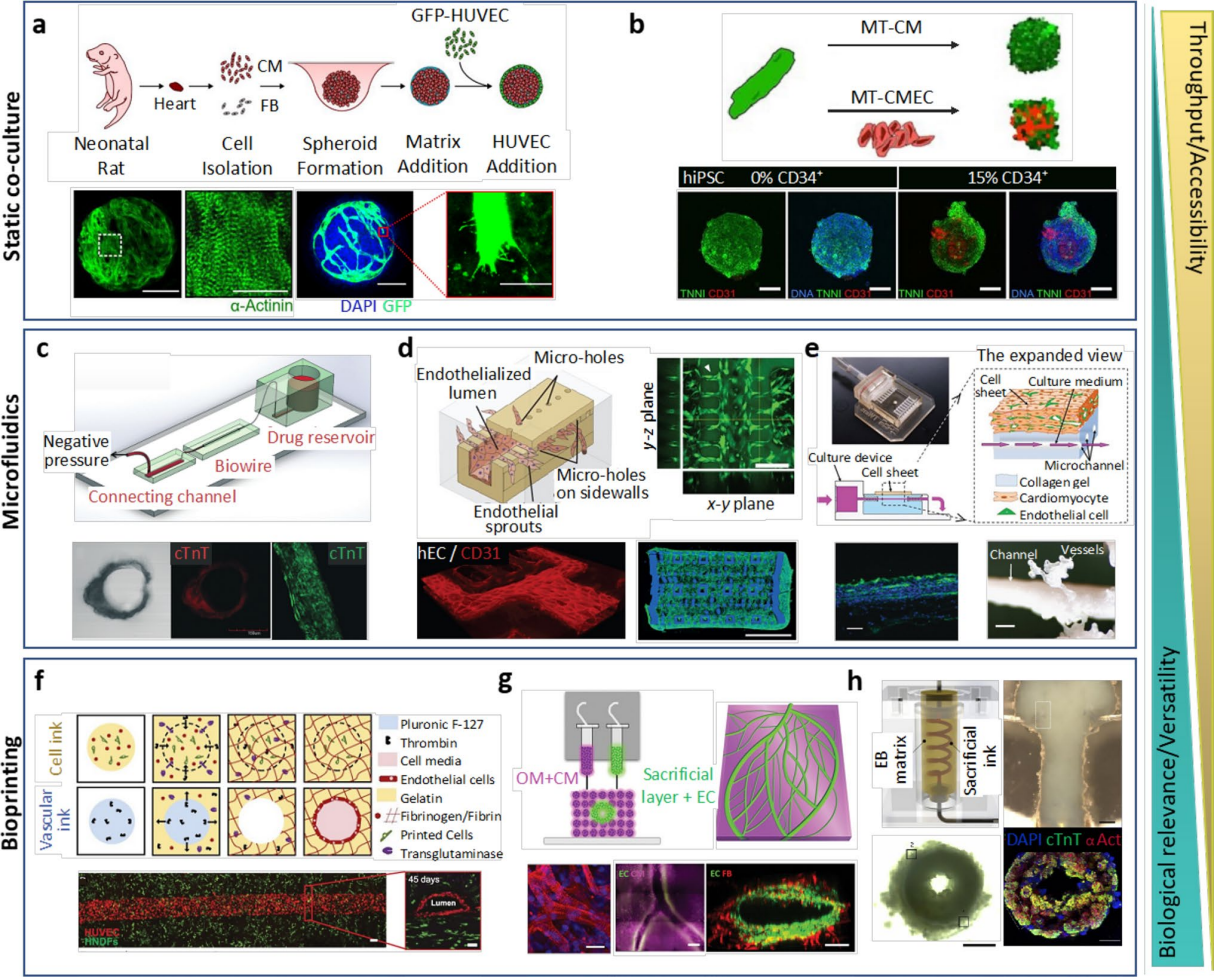
In vitro models of myocardial-microvascular interaction. (**a**) Spheroid based static co-culture of cardiomyocytes (CM), fibroblast (FB), and human umbilical vein endothelial cells (HUVEC) [194]. (**b**) Co-differentiated human-induced pluripotent stem cell–derived cardiomyocytes (hiPSC-CM)-endothelial cell (EC) 3D microtissue (MT) [47]. (**c**) Microfluidic Biowire setup used for creation of perfused cardiomyocyte bundle [202]. (**d**) Microfluidic Angiochip designed to create branching vascular network and endothelialised multi-layer cardiac tissue [212]. (**e**) Cell sheet technology combined with a perfusion bioreactor creating a vascularized thick tissue [166]. (**f**) Multi-material extrusion-based bioprinting technique creating thick tissue with vascular lumen [88]. (**g**) Bioprinted patient-specific thick, perfused, and vascularized cardiac patch made from omental tissue (OM) [136]. (**h**) Embryoid body (EB)– based bioprinted cardiac tissue using SWIFT method [175]

Encapsulation in 3D biomaterials is one of the most technically accessible and cost-effective ways to remove cells from rigid tissue culture plastic and provide them with a more familiar biomechanical microenvironment [23]. Discussion of different biomaterial types is beyond the scope of this review, but many natural and synthetic options are available, allowing researchers to tailor microenvironments to an application of choice [153], e.g. tuneable stiffness [100] or promotion of vascularisation [18, 73]. 3D encapsulation not only provides a biomimetic mechanical substrate but also gives cells the mobility required to spontaneously reorganise into physiologically relevant architectures. This has proven particularly valuable in elucidating the biochemical and biophysical nature of endothelial vessel formation [125, 216]. 3D encapsulation has also been utilised to better understand myocardial heterocellular interactions. In 2007, Caspi et al. [24] published the first “vascularised” in vitro cardiac cultures. By combining hESC-CM, human umbilical vein endothelial cells (HUVEC), and embryonic fibroblasts (EmFs) in a porous sponge scaffold (composed of 50% poly l-lactic acid and 50% polylactic glycolic acid), the authors observed spontaneous assembly of EC networks with open luminal spaces. Incorporation of FBs or other stromal cells (e.g. pericytes, mesenchymal stem cells) as a source of angiogenic/vasculogenic growth factors is a common strategy for inducing vessel assembly in in vitro vascular tissue engineering [68, 131]. However, in the developing heart and during hypertrophy, CM are the primary regulators of de novo vessel formation [195]. While iPSC-CM have been shown to produce angiogenic extracellular vesicles, whether they can fully coordinate mature vessel formation as their native counterparts do, is yet to be shown. A recent neonatal rat ventricular (NRVM) model, based on hanging droplet “cardiac tissue mimetics” (CTM), is an example of a potential 3D in vitro platform to study iPSC-CM angio-/vasculogenic capacity [194] (Fig. 2a). This approach generated a 3D microtissue containing NRVM and FB, and after 3 days added HUVEC. The authors observed angiogenic NRVM-HUVEC communication, illustrating enhanced endothelial network formation in the presence of alpha-1 receptor agonist phenylephrine.

An important consideration when designing in vitro models is the source of non-myocyte cells. A great benefit of iPSC-based models is the capacity to study human biology, so an ideal model would also contain human non-myocytes from a relevant tissue. Human ECs isolated from different tissues maintain phenotypic and behavioural differences after isolation and in vitro culture [113]. When comparing fetal ECs from heart, lung, liver, and kidney after five in vitro passages, heart ECs displayed significantly higher trans-endothelial electrical resistance, angiogenic potential, and metabolic rate [113]. Indeed, heterogeneity is also present amongst EC isolated from different parts of the heart, with EE and MVEC displaying distinct phenotypes in vivo, including expression of receptors for natriuretic peptides [200], angiotensin-converting enzyme [204], endothelin subtypes [124], mineralocorticoids [107], major histocompatibility complexes [144], and adhesion molecules [144, 181]. With growing commercial availability of human cardiac non-myocytes, it seems clear that cardiac ECs should be used when possible. Going one step further, CM-EC crosstalk in myocardial morphogenesis was modelled by Giacomelli et al., who co-differentiated both CM and EC from the same human iPSC line, before combination in 3D microtissues [47] (Fig. 2b). While the structure and function of iPSC-derived EC (iPSC-EC) are still considered immature compared to primary EC, and differentiation protocols for specific EC subtypes are a work in progress [71], EC differentiated from iPSC is a strategy which may prove useful for patient-specific applications, such as disease modelling and regenerative medicine.

In 3D biomaterial-based cultures, deformable substrates allow encapsulated EC to experience cyclical mechanical stimulation as neighbouring CM beat. While this represents a significant move towards the biomechanics of the myocardium, a key limitation of static models is the absence of the haemodynamic stimuli which ECs require to instruct their behaviour in vivo. While EC networks are observed in 3D static cultures, without the presence of an open luminal space with continuous fluid flow, these structures cannot be considered functional vessels. With that being said, the fields of microfluidics and more recently bioprinting have enabled development of models which drive us closer towards functional myocardial vasculature.

### Perfused methods—microfluidics

In recent decades, the application of microfluidics to biological experimentation has produced the concept of an organ-on-a-chip (OOC), a micro-scale replicate of in vivo tissue [10]. Microfluidic pumps connected to OOCs subject 2D or 3D cultures to precisely control media perfusion and haemodynamic forces, enabling the reproduction of biomimetic flow profiles. For vascular tissue engineering, microfluidic models have allowed delivery of well-defined physical and biochemical stimuli (angiogenic growth factors) to induce spontaneous assembly of human ECs into fully perfusable capillary networks [128, 154]. Such microvasculature is highly biomimetic, compatible with high-resolution microscopy for haemodynamic analysis, and can be combined with organ- or disease-specific parenchymal cells to model specific microdomains, i.e. tumour microenvionment [72]. This spontaneous assembly of vasculature can be considered a “bottom-up” approach, in which vascular structures are designed by the cells, not predefined by the user. The result of giving creative freedom to the cells is randomly oriented capillary beds. However, some control of vessel orientation can be achieved by regulating environmental factors such as flow direction [83] and growth factor gradients [172]. Whether these “bottom-up” strategies will be effective in the densely packed, mechanically dynamic myocardium is yet to be determined. “Top-down” strategies, in which a vessel structure is built by the user, can be achieved by many different microfabrication techniques, as recently reviewed by Tomanasina et al. [186]. This grants more control over vessel orientation, but is limited by the resolution of the microfabrication technique, and is therefore is suited to larger vessels rather than capillaries. As such, the branch of the coronary tree which the user wishes to model will influence the choice of technique.

However, physiological relevance is not the only incentive to design perfused models. Regulated perfusion of cultures allows for control (or automation) of the extracellular chemical environment and drug delivery [25], which, when coupled with scalability for high throughput, presents perfusable cardiac microfluidic models as a valuable asset for the pharmacological industry to perform screening of novel therapeutics [162]. As such, some early myocardial microfluidic models aimed squarely at this output, with platforms that could expose multiple replicates of cardiac myocytes (typically NRVMs or iPSC-CM) to perfusing solution while optically assessing functional contractile parameters, such as substrate deformation [2], conduction velocity [116], or beating rate [14]. While this approach is not fully biomimetic, as myocytes in the heart are not exposed to haemodynamics, these models illustrate the technical possibility of incorporating precisely controlled fluid flow in in vitro cardiac models. Qian et al. recently delegated flow control to beating iPSC-CM by recording contractile waveforms from cardiac spheroids, then using the pulsatile waveform to control the flow velocity of a pump connected to endothelial monolayers [156]. Though this model does not culture CM and EC together, and uses 2D EC culture rather than vessels, by using iPSC-CM generated from patients with long-QT syndrome, and treating the spheroid with verapamil and nifedipine, it demonstrates an innovative method of investigating physiologically and pathophysiologically relevant flow profiles on EC function in real time.

Other microfluidic strategies have put more emphasis on replicating physiological structures. The cardiac Biowire technology described by Nunes et al. [138] mimics the structural organisation of a capillary within a cardiac bundle, by surrounding a perfusable channel with a 3D hPSC-derived cardiac tissue (Fig. 2c). Though of lower throughput than the previously mentioned microfluidic models, this technology represents an in vitro advance towards the true physiological association between cardiac myocytes and the circulation [202]. In a microfluidic chip, Ellis et al. modelled the cardiac endothelium by seeding 2D monolayers of iPSC-EC on the surface of perfusion channels which deliver media to a 3D CM culture. Though CM and EC are not physically in contact in these chips, perfusing media comes into contact with a living endothelium before reaching CM, mimicking native physiology where flow and nutrients approach the myocardium via an endothelium [36]. Another interesting model, designed with the intention of in vivo transplantation, is the AngioChip [211, 212] (Fig. 2d)*.* This technique employs 3D patterning of a biodegradable polymer to form a series of perfusable channels in which endothelial monolayers are seeded. An ECM/parenchymal cell (both cardiac and hepatic models are demonstrated) matrix is then seeded on the opposite side of the endothelialised channels. While ECs are physically separated from CM (and thereby not experiencing contractile biomechanical stimulation) micropores in the polymer structure allow cell migration and paracrine interaction between endothelium and parenchyma. All of these models represent significant advances towards in vitro reproduction of the myocardial-microvascular interface under biomimetic perfusion.

A long-time tissue engineering hurdle has been delivery of nutrients to 3D cultures thicker than the diffusion limit. iPSC-based cardiac tissue engineering has the potential to replace damaged myocardium [215], but to produce cardiac tissue in vitro of clinically relevant size, vascularisation is necessary to deliver nutrients throughout the culture to prevent formation of a necrotic core [157]. To tackle this issue, Sekine et al. induced vascularisation in 3D CM/EC cell sheets layered on top of a resected section of femoral tissue [90]. The authors observed anastomosis of femoral tissue vessels and de novo vessels from the overlaid cell sheet co-culture, resulting in a vascular network amongst beating myocytes. This experimental approach illustrates the potential of spontaneous endothelial activity by inducing a mixture of angiogenesis (generation of new vessels from an existing vessel) from the femoral tissue, and vasculogenesis (generation of new vessels without a pre-existing vessel). Subsequent work subverted the need for an animal donor by replacing the femoral tissue with a collagen gel containing microchannels, through which angiogenic growth factors (VEGF, FGF) were perfused [166] (Fig. 2e). With this approach, the authors observed migration of cells into the microchannels, formation of vessels within the overlaid cell sheets, and viability of tissues up to 100 µm thick. However, the apparatus required to generate these models are highly specialised and limit their combination potential observational techniques.

Meanwhile, there is great potential for integration of optical measurements and biochemical sensors in microfluidic chips [38], allowing real-time evaluation of structure and function of both the CM and EC under hemodynamic forces. An example of this is simultaneous observation of EC barrier function (via trans-endothelial electrical resistance (TEER) measurement) and myocardial conduction velocity (via multi-electrode array (MEA)) [112]. With this method, the ability to detect dynamic alterations of vascular permeability and cardiac function within the same chip when perfused tumour necrosis factor alpha (TNF-α) or isoproterenol was shown, thereby having the ability to gain spatially and temporally resolved information in real time. Lind et al. demonstrated the potential of micro-patterning sensors in 3D using 3D printing [104]. This work printed piezo-resistive, high- conductance, and biocompatible soft materials that enable integration of soft strain gauges for measurement of cell contractility. Shiwarski et al. recently described a soft, biocompatible strain sensor made from a fluorescently labelled fibronectin mesh [174]. Given that fibronectin is a naturally occurring component of myocardial ECM, and of particular importance to post-MI repair [91], such bio-inspired sensors would enable non-invasive measurement of cellular biomechanics with minimal disruption to cell behaviour. As mentioned in the first section of this review, evaluation of myocardial microvascular biology has been hampered the inability to combine quantitative experimental techniques with the complex experimental platforms required to visualise microvasculature in the beating myocardium (i.e. open heart transillumination microscopy). As such, the capacity for sensor integration and automated data acquisition and analysis in human OOCs has great potential to increase the insight and experimental value of in vitro myocardial models.

### Perfusable methods—bioprinting

Bioprinting is a technique which allows fabrication of complex 3D geometries composed of cells and biomaterials [129]. Unlike many microfluidic technologies, bioprinting enables production of heterocellular cultures with complex 3D architectures distributed throughout one continuous biomaterial, i.e. no rigid support structures separating cells [191]. In a fundamental demonstration of the potential for bioprinting to fabricate vascular structures, Kolesky et al. described an extrusion-based multi-material 3D printing technique that simultaneously patterns acellular or cell-laden “bioink” hydrogel filaments [89]. In this technique, a bioink with reversible gelation serves as a sacrificial material which is removed after printing, leaving behind an open lumen [89] (Fig. 2f). Perfusion of these preparations resulted in fully functional HUVEC-based vessels embedded in a 3D FB microtissue [88]. This illustrates the capacity of bioprinting to produce in vitro vessels which expose the luminal side of polarised ECs to haemodynamics, while providing a supporting ECM for the abluminal side to anchor to. While OOC’s and microfluidic models typically rely on rigid polymers or glass as support structures to contain the tissue, bioprinted models can be produced entirely with deformable, mechanically biomimetic materials. This was beautifully illustrated when Grigoryan et al. produced a model of the lung with fully perfusable alveolar geometries embedded in a cytocompatible poly(ethylene glycol) hydrogel, which could be mechanically actuated to simulate tidal ventilation [52]. The biomechanical relevance of these models to cardiac biology are obvious and represent an exciting avenue for future work.

Another benefit of bioprinting is that patient-derived imaging data can be used as a blueprint for the model, allowing cardiac researchers to tackle the ambitious aim of recapitulating macro- and microscale cardiac anatomy in vitro. Noor et al. generated a model of the vascularised left ventricle by using computerized tomography (CT) to identify the 3D structure and orientation of major blood vessels, combined with a mathematical model optimised for oxygen diffusion and consumption to define the layout of smaller vessels [136] (Fig. 2g). Printed vessels were embedded in iPSC-CM sheets, resulting in highly biomimetic physical association between myocardium and vasculature. This model also demonstrated impressive potential for patient specificity, by using patient-derived omental ECM as the supporting biomaterial, populated by patient-derived iPSC-CMs and iPSC-ECs. This work serves as a proof of principle that a patient-specific, anatomically complex tissue of clinically relevant scale can be produced. However, key limitations of this study are lack of controlled flow through printed vessels and restriction to macro-scale vessels. Skylar-Scott et al. developed a perfusable printing technique called SWIFT (sacrificial writing into functional tissue) which prints sacrificial vessel structures in a slurry of prefabricated densely packed iPSC-CM organoids [175] (Fig. 2h). These preparations are perfusable, and, in separate non-cardiac experiments, the authors demonstrate endothelialisation via post-printing seeding of EC within the channel lumens. This approach therefore has the potential to produce 3D iPSC-CM cultures, in biomimetic ECM, with a perfused vessel containing polarised ECs, thereby allowing for both contractile and haemodynamic biomechanics. However, vessel diameter is again a limitation, with minimum diameters being 400 µm. Considering myocardial capillaries can be as small as 5–10 µm, which is far below the typical diameter of a printer extrusion needle, resolution is a prevalent issue for most bioprinting techniques aiming to reproduce micro-scale structures [97].

However, recent development of FRESH (freeform reversible embedding of suspending hydrogels) by Lee et al. demonstrated bioprinting resolution of 10 µm [96]. This is a highly adaptable bioprinting technique which is compatible with a variety of bioink materials, support bath materials, crosslinking chemistries, and print pathing modes [173]. Lee et al. illustrate the potential of this technology by producing perfusable (though acellular) multi-scale vasculature trees, ranging from coronary vasculature size down to 100-um diameter. This technique relies on collagen self-assembly forming a porous microstructure, which was demonstrated to be suitable for cellular infiltration and spontaneous micro-vascularisation in vivo. Coupling spontaneous microvascular angiogenesis/vasculogenesis with larger printed vessels will likely be most effective strategy to reproduce both macro- and microvascular coronary vessels.

Encouragingly, bioprinting of multi-scale vascular networks has been done [98]. Lee et al. printed acellular channels with 1-mm lumens, separated by a fibrin hydrogel containing HUVEC and lung FB. HUVEC were subsequently seeded in the printed channels. Spontaneous angiogenesis from the large vessels, and vasculogenic capillary bed formation in the fibrin gel resulted in anastomosis of large and small vasculature. Myocardial microvascularisation through printing could also be achieved by printing the cellular and ECM precursors required to promote spontaneous vasculogenesis (described in Sect. 4.1), yet this will require a balancing act of many physical and chemical factors [66]. One potential hurdle specific to bioprinting cardiac tissue with relevant biomechanics is cell density. The high density of CM in the compact myocardium required for propagation of contractile forces may disrupt biomaterial stoichiometry and gelation during printing [214]. Future work will need to unravel the printing materials and experimental logistics which satisfy both EC and CM viability while promoting spontaneous vasculogenesis and maintaining the biomimetic CM density required for physiologically relevant biomechanics.

In summary, bioprinting is an advanced technique which has demonstrated impressive capacity to produce complex biomimetic architectures. This method is still highly specialised and limited by technical complications such low resolution and integration with controlled perfusion, meaning only a handful of studies have thus far demonstrated its potential to produce biomimetic vascularised myocardium. However, recent reviews on innovative techniques for printing microvasculature-scale architectures [12] and integration of bioprinting with the controlled perfusion of microfluidics [118, 122, 178] illustrate the rapid pace of development in this exciting field. Given time for the technology to mature and become more accessible, bioprinting holds strong potential to become a widely adopted method of generating physiologically relevant human models of the vascularised myocardium.

## Conclusions and future prospects

The interaction between CM and MVEC occurs in a biomechanically dynamic environment. Though microvascular dysfunction is now recognised as a prevalent driver of cardiac disease, in vitro models which can replicate the biomechanics and 3D vascular architectures of the beating myocardium are limited. However, the development of CM differentiation protocols from PSCs and novel tissue engineering fabrication techniques are facilitating production of functionally vascularised human in vitro myocardial models of unprecedented physiological relevance. By supporting cardiac contractile and haemodynamic biomechanics, these models promise to enable investigation into previously inaccessible cardiac phenomena, such as the role of biomechanical stimuli as regulators of CM-EC crosstalk in health and disease. In particular, a physiologically relevant human in vitro model which would allow investigation into the contribution of microvascular dysfunction to pathologies such as heart failure and ischaemic heart disease is of pressing importance. A unique and powerful benefit of PSC based models is the ability to genetically modify human tissue. While opening up the possibility to model heritable disorders [119], gene editing can also enhance the experimental value of a cell model though genetic encoding of reporters which provide real- time analysis of function, i.e. fluorescent labelling of sarcomeres [171] or expression of calcium indicators [67]. Incorporation of physically relevant biomechanical conditions can be achieved through use of deformable 3D biomaterial substrates, and perfusable microfluidic and bioprinted cultures. The experimental versatility of microfluidic platforms and OOCs is particularly high, with models incorporating integrated and automated sensing systems to monitor metabolic activity, oxygen levels, electrophysiology, and mechanical activity [38]. The combination of integrated sensing and precisely controlled flow conditions will allow researchers to dissect the role of biomimetic haemodynamics in myocardial-microvascular interaction. Bioprinting offers an even greater degree of 3D complexity, with current technology using mechanically biomimetic substrates to build sophisticated anatomical structures such as the coronary vascular tree [96], valves [96], and whole heart models [62, 121]. Advances in stem cell–based tissue engineering techniques are therefore moving us ever closer to vascularised human myocardial models with biomechanics approximating those of the human heart, offering exciting opportunities for new insights into human cardiac biology.

## Supplementary Information

Below is the link to the electronic supplementary material.

Supplementary file1 (MP4 1184 KB)
